# Enrichment of White Chocolate with Microencapsulated β-Carotene: Impact on Quality Characteristics and β-Carotene Stability during Storage

**DOI:** 10.3390/foods13172699

**Published:** 2024-08-26

**Authors:** Christina Drosou, Magdalini Krokida

**Affiliations:** School of Chemical Engineering, National Technical University of Athens, 9 Heroon Polytechniou St., Zografou Campus, 15780 Athens, Greece; mkrok@chemeng.ntua.gr

**Keywords:** β-carotene, electrospinning process, encapsulation, freeze drying, spray drying, white chocolate

## Abstract

This study developed functional white chocolate enriched with free (WC-F) and encapsulated β-carotene using whey protein isolate (WPI) and pullulan (PUL) blends through spray drying (WC-SP), freeze drying (WC-LP), and coaxial electrospinning (WC-EL). The thermal properties, rheological properties, hardness, and color of the chocolates were evaluated, and the stability of β-carotene was monitored over 4 months at 25 °C. No significant differences were found in melting profile temperatures among samples; however, WC-LP and WC-EL exhibited higher melting energies (30.88 J/g and 16.00 J/g) compared to the control (12.42 J/g). WC-F and WC-SP showed rheological behaviors similar to those of the control, while WC-LP and WC-EL displayed altered flow characteristics. Hardness was unaffected in WC-F and WC-SP (7.77 N/mm^2^ and 9.36 N/mm^2^), increased slightly in WC-LP (10.28 N/mm^2^), and decreased significantly in WC-EL (5.89 N/mm^2^). Over storage, melting point, rheological parameters, and hardness increased slightly, while color parameters decreased. β-carotene degradation followed a first-order reaction model, with degradation rate constants (*k*) of 0.0066 day^−1^ for WC-SP, 0.0094 day^−1^ for WC-LP, and 0.0080 day^−1^ for WC-EL, compared to 0.0164 day^−1^ for WC-F. WC-SP provided the best β-carotene retention, extending the half-life period by 2 times compared to WC-F (126.04 days vs. 61.95 days). **Practical implications:** The findings suggest that WC-SP, with its superior β-carotene stability, is particularly suitable for the development of functional confectionery products with extended shelf life, offering potential benefits in industrial applications where product stability is crucial. **Future research directions:** Further studies could explore the incorporation of additional bioactive compounds in white chocolate using similar encapsulation methods, as well as consumer acceptance and sensory evaluation of these enriched products.

## 1. Introduction

Chocolate is a complex blend of various components, including 65–75% solid particles such as sugar, cocoa, and milk powder, within a continuous fat phase, typically cocoa butter and milk fat, depending on the type of chocolate [1]. Cocoa, one of the key components of plain chocolate, is rich in flavonoids, epicatechin, flavanol, procyanidin, etc., which have beneficial effects on human health due to their antioxidant properties [2]. Notably, polyphenol content in chocolates is not consistent across all varieties. Elevated polyphenol content is commonly observed in dark chocolate, distinguished by its higher cocoa content compared to milk chocolate [3]. In contrast, white chocolate, despite lacking the antioxidant-rich cocoa solids found in dark chocolate, remains a popular confectionery product enjoyed by consumers worldwide due to its creamy texture and sweet flavor. However, its composition also makes it a prime candidate for functional enhancement, particularly through the incorporation of bioactive compounds, as it lacks the polyphenols found in cocoa, resulting in a deficiency of natural antioxidants [4]. Therefore, there is a need to enhance the antioxidant activity of white chocolate and enrich it with bioactive components.

Currently, the trend in the food industry involves the development of confectionery products enriched with bioactive compounds. This is driven by the increasing demand for functional foods that offer health benefits. Various bioactive compounds such as fatty acids, carotenoids, tocopherols, polyphenols, phytosterols, fat-soluble vitamins, and probiotics are being explored as potential enrichment agents for chocolate [5]. However, studies related to the development of functional foods involving white chocolate are limited. Specifically, Maillard and Landuyt (2008) studied the development of probiotic dark, milk, and white chocolate by incorporating encapsulated probiotics *Lactobacillus helveticus* and *Bifidobacterium longum* in combination with fatty acids through spray drying [6]. Gil-Ramírez et al. (2014) investigated the hypocholesterolemic properties of white chocolate enriched with ergosterol from *Agaricus* [7]. Poliński et al. (2022) investigated the influence of bioactive compounds from powdered leaves of matcha green tea (*Camellia sinensis* L.) and moringa (*Moringa oleifera*) on the antioxidant properties of white chocolate during its processing [8]. Furthermore, Belščak-Cvitanović et al. (2012), and Lončarević et al. (2018, 2019) studied the enrichment of white chocolate with polyphenols derived from red raspberry leaves (*Rubus idaeus* L.), black and green tea extracts, and blackberry juice, respectively [9,10,11]. Finally, the development of white chocolate with incorporated EPA and DHA fatty acids and pigments from the microalga *Nannochloropsis oculata* was reported in studies by Genc Polat et al. (2020) and Toker et al. (2018), respectively [12,13].

The incorporation of β-carotene in the development of functional foods has garnered significant interest in various research studies within the field of food technology. β-carotene stands out as one of the primary types of carotenoids, exhibiting robust biological activity. It is commonly employed in the food industry, serving either as a precursor to vitamin A or as a natural pigment [14]. However, the effectiveness of β-carotene relies on preserving its stability, as it tends to degrade easily during food processing and storage, being sensitive to factors such as heat, light, and oxygen. Moreover, its vibrant color imposes limitations on the types of foods into which it can be seamlessly integrated [15]. Consequently, β-carotene is encapsulated in various biopolymers to protect it from water, oxygen, and light, ultimately facilitating its optimal incorporation into food systems [16]. In the available literature, numerous studies have delved into the incorporation of encapsulated carotenoids into various food systems. For instance, encapsulated β-carotene in almond gum and gum arabic was utilized in the production of cakes, with results indicating a significantly higher a* value in the cake containing almond gum compared to the one with gum arabic [17]. These outcomes may be attributed to the superior protection of encapsulated β-carotene in almond gum compared to gum arabic. In another study, Kha et al. (2015) explored the development of yogurt, pasteurized milk, and cake with encapsulated Gac oil in whey protein and gum arabic. Findings revealed that the color of products with incorporated Gac oil remained stable, while there was a slight decrease in β-carotene and lycopene during storage [18]. Furthermore, Coronel-Aguilera and Martin-Gonzalez (2015) studied the development of yogurt enriched with encapsulated β-carotene in maltodextrin and sodium caseinate, comparing the product with commercial peach yogurt. The results indicated that the overall color value of the yogurt manufactured with encapsulated β-carotene was comparable to the standard value and remained stable in acidic conditions for 4 weeks of storage at 4 °C [19]. However, to the best of our knowledge, there are no reports regarding the incorporation of β-carotene into chocolate products.

Traditional encapsulation methods such as spray drying and freeze drying have been widely adopted in the food industry for the incorporation of bioactive compounds into various food matrices. These techniques are well-established and offer practical benefits, such as scalability and cost-effectiveness, making them suitable for large-scale production [20]. Spray drying, for instance, is frequently used for the encapsulation of heat-sensitive ingredients due to its ability to produce stable, dry powders. Freeze drying, on the other hand, is known for its capability to preserve the integrity of bioactive compounds by removing water through sublimation under low temperature conditions. However, these conventional methods also have limitations, particularly in terms of particle size control and encapsulation efficiency, which can affect the stability and retention of the encapsulated compounds during storage [21,22,23]. In contrast, coaxial electrospinning emerges as an innovative technique that offers precise control over the encapsulation process, allowing for the creation of nanofibers with a core-shell structure [20,24]. This structure provides superior protection to sensitive bioactives, such as β-carotene, against environmental factors like oxygen, light, and heat, thereby enhancing their stability during processing and storage. The novelty of coaxial electrospinning lies in its ability to produce encapsulated structures with unique physicochemical properties that are difficult to achieve with traditional methods [25]. Despite the promising potential of coaxial electrospinning, there is currently a limited body of research comparing its effectiveness directly with conventional encapsulation techniques like spray drying and freeze drying. This gap underscores the need for further studies to determine the most suitable encapsulation method for specific applications in food systems, particularly for the development of functional foods.

The aim of this study was to develop functional white chocolate enriched with encapsulated β-carotene using blends of WPI and PUL, enhancing stability during processing and storage while preserving qualitative characteristics. A comparative evaluation was conducted on white chocolate incorporating free β-carotene and β-carotene encapsulated through conventional methods such as spray drying and freeze drying, alongside the emerging coaxial electrospinning process. The evaluation focused on the impact of incorporating β-carotene encapsulants on the qualitative characteristics of white chocolate, including thermal properties, rheological properties, hardness, and color. Finally, the stability of β-carotene in white chocolate products with different encapsulation structures was studied over a 4-month period at ambient temperature.

## 2. Materials and Methods

### 2.1. Materials

For white chocolate production, milk powder (0.2% fat content), cocoa butter, sugar, lecithin, and vanilla were kindly donated from Giotis S.A. (Athens, Greece). WPI and PUL (IP 20) were supplied by NOW^®^ SPORTS (Athens, Greece) and Hayashibara Biochemical Lab. Inc. (Okayama, Japan), respectively. β-carotene (Type I, synthetic, ≥93% purity (UV), powder) and analytical grade chemicals were supplied by Merck SA (Athens, Greece).

### 2.2. White Chocolate Production

The production of white chocolate was carried out in the Cocoa T Deluxe Melanger ECGC-12SLTA/12SL (CocoaTown LLC, Alpharetta, GA, USA). The recipe for white chocolate included sugar (47.00%), cocoa butter (31.00%), milk powder (21.50%), soy lecithin (0.49%) and vanilla extract (0.01%). The melted cocoa butter (comprising 70% of the total cocoa butter quantity), milk powder, and sugar were mixed until a homogeneous mixture was formed while being heated to 40 °C. Mixing and refining took place over the first 3 h, resulting in a mass with a smooth texture and a particle size of approximately 28–30 μm. Dry conching followed, with a simultaneous increase in temperature to 60 °C for 3 h. Then, the remaining cocoa butter was added, and the mixture was processed for 6 h at 65 °C. Subsequently, wet conching took place for 6 h at 40–45 °C. Two to three hours before the end of conching, the emulsifier (lecithin) and flavoring ingredients (vanilla) were added. Subsequently, the tempering procedure took place. Chocolate was transferred to a glass container at 50 °C. Two-thirds of the chocolate was transferred to a cold marble slab, where it was continuously rubbed for approximately 10 min to reach a temperature of 18–22 °C, stabilizing the β-crystals. The chocolate was then mixed with the remaining one-third of the warm chocolate and stirred to bring the temperature back to 27–30 °C, melting any crystals that were not in the β-form. Finally, after tempering, the chocolate was poured into plastic molds with dimensions of 3 cm in length and 2 cm in height and chilled in a refrigerator at 4 °C for 1 h before demolding. Subsequently, the chocolates were arranged on plastic trays and stored at 25 °C.

### 2.3. Development of Encapsulated β-Carotene Structures Using Electrospinning Process, Spray Drying and Freeze Drying

To evaluate the effectiveness of different encapsulation techniques for preserving β-carotene in white chocolate, three distinct processes, including the electrospinning process, spray drying, and freeze drying, were employed using a WPI: PUL polymeric blend serving as the carrier material. Each method was selected for its unique properties. Spray drying is commonly used in the food industry due to its efficiency and scalability in producing stable dry powders, making it particularly relevant for commercial applications. Freeze drying, on the other hand, was chosen for its ability to maintain the structural and bioactive integrity of sensitive compounds, which makes it especially effective in preserving β-carotene during the encapsulation process. Lastly, coaxial electrospinning was included as an innovative technique that allows for the formation of nanofibers with a protective core-shell structure, offering enhanced stability against environmental degradation. This novel approach was selected to explore its potential advantages over traditional methods. The development of the encapsulated structures was accomplished under optimal experimental conditions in terms of β-carotene encapsulation efficiency, based on our previous studies [21,25].

#### 2.3.1. β-Carotene Electrospun Structures

Coaxial electrospinning for the development of β-carotene electrospun structures (EL) was carried out using the FluidNatek^®^ electrospinning apparatus (BioInicia S.L., Paterna, Spain). The carrier solution, consisting of a WPI:PUL polymeric blend (30:70 weight proportion, 20% *w*/*w* concentration), and the core solution, containing β-carotene in corn oil (0.25 mg/mL concentration), were processed under the following conditions: carrier solution flow rate of 2.0 mL/h, β-carotene solution flow rate of 0.1 mL/h, applied voltage at 19 kV, and tip-to-collector distance of 17 cm. The composition of the carrier solution was selected based on our previous studies [24,25]. For the coaxial electrospinning process, a higher proportion of pullulan was chosen to enhance spinnability, which is critical for forming uniform and continuous core-shell structures. Pullulan provides better viscosity and polymer chain entanglement, which are necessary for producing stable structures. This adjustment in the ratio also enhances the protective properties of the structures, thereby ensuring better encapsulation and stability of β-carotene.

#### 2.3.2. β-Carotene Spray-Dried Structures

For the spray drying and freeze drying processes, a WPI:PUL ratio of 80:20 *w*/*w* was selected based on our previous study [21]. This ratio was optimized based on emulsifying properties of WPI, which are essential for creating stable encapsulated structures through these drying techniques. The higher proportion of WPI ensures efficient encapsulation while maintaining the bioactive integrity of β-carotene during the drying processes.

The production of β-carotene spray-dried structures (SP) involved using a parallel flow spray dryer (Model YC-015, Shanghai Pilotech Instrument & Equipment Co., Ltd., Shanghai, China). A stable oil-in-water emulsion was prepared with a WPI:PUL polymeric blend at a concentration of 6.0% *w*/*w*, maintaining a consistent weight proportion of 80:20 *w*/*w* for the two materials and 5% *w*/*w* of the dispersed oil phase (0.25 mg β-carotene/mL corn oil). The operating conditions were set with an emulsion flow rate of 500 mL/h and an inlet temperature of 170 °C.

#### 2.3.3. β-Carotene Lyophilized Structures

The development of β-carotene lyophilized structures (LP) was accomplished using a freeze dryer (Leybold-Heraeus GT 2A, Koln, Germany) operating at a pressure of *p* = 0.2 mbar for 48 h. Similarly with Sub-Section 2.3.2, an oil-in-water emulsion was prepared using WPI:PUL polymeric blend at a concentration of 8.0% *w*/*w*, maintaining a constant weight proportion of 80:20 *w*/*w* for the two materials and 5% *w*/*w* of the dispersed oil phase (0.25 mg β-carotene/mL corn oil).

### 2.4. Incorporation of Encapsulated β-Carotene Structures in White Chocolate

White chocolate was selected as a model system for the encapsulated β-carotene structures. The following white chocolates were prepared: (i) white chocolate control (WC-C), (ii) white chocolate with free β-carotene (WC-F), (iii) white chocolate with incorporation of β-carotene EL (WC-EL), (iv) white chocolate with incorporation of β-carotene SP (WC-SP), and (v) white chocolate with incorporation of β-carotene LP (WC-LP). The β-carotene concentration in white chocolate was standardized to 1.0 mg/100 g. Based on the β-carotene loading capacity of the encapsulated structures, they were incorporated into the chocolate at the following concentrations: 2.67%, 0.67%, and 7.50% *w*/*w* for EL, SP and LP, respectively, to maintain a consistent β-carotene concentration across all formulations. The β-carotene EL and β-carotene LP were ground before incorporation into the chocolate system. The addition of β-carotene occurred during the chocolate production process at the stage of incorporating emulsifiers and flavoring agents to prevent oxidation.

### 2.5. Thermal Properties

The melting properties of white chocolate were determined using a differential scanning calorimeter (Pyris DSC-6, Perkin Elmer Ltd., Shelton, CT, USA). Samples (approximately 20–40 mg) were loaded into sealed aluminum pans, with an empty pan used as a reference. The pans were tempered at 0 °C for 2 min and then heated from 0 °C to 55 °C at a constant rate of 5 °C/min. Nitrogen gas was used to create an inert atmosphere at a flow rate of 20 L/min. Onset temperature (*T*_onset_), end temperature (*T*_end_) peak temperature (*T*_peak_: melting temperature) and enthalpy of melting (∆*H*_m_) were calculated with the Pyris 6 software version 4.01. All measurements were performed in triplicate to ensure reliability and reproducibility.

### 2.6. Rheological Properties

Rotational rheological measurements of the molten white chocolates were performed using a Physica MCR 301 rheometer (Anton Paar, Ostfildern, Germany) with plate-plate geometry (2 mm gap). Chocolate samples were prepared by heating in an oven at 45 °C for 1 h to achieve a molten state. Approximately 2–4 g of the melted chocolate was then applied to the plate, and the samples were tempered at 40 °C for 2 min. Shear stress was recorded while varying the shear rate from 0.1 to 100 1/s. All the tests were performed at 40 °C. Shear rate (1/s) and shear stress (Pa) were recorded electronically using Rheoplus/32 V3.40 software. The data were fitted to the Casson model, with the Casson yield stress (σ) calculated as the square of the intercept and the Casson plastic viscosity as the square of the slope ($\dot{\gamma }$). All measurements were conducted in triplicate to ensure accuracy and reproducibility.

### 2.7. Hardness

The hardness of white chocolate was determined using uniaxial compression tests in a universal testing machine (Zwick model Z2.5/TN1S, Ulm, Germany). These tests were performed at room temperature with a 2000 N load, employing a constant deformation rate of 5 mm/min for all samples. Compression tests were stopped when the force reached its maximum value. The samples were cylindrical in shape, and their dimensions were measured prior to each experiment using a Vernier caliper. Force and deformation were electronically recorded using specialized software (Zwick PC Software, Version 3.1). Stress–strain compression curves were constructed using the recorded data and the provided equations:

(1) $$\sigma =\frac{F}{A},$$

(2) $$\varepsilon_{n}=\frac{∆L}{L_{o}},$$

where, *σ* is the stress (Pa), *ε_n_* is the strain (mm/mm), *A* is the cross-section area (m^2^), *L_o_* is the initial thickness of the samples (m), *F* is the force (N), and ∆*L* is the deformation (m). The elasticity parameter (*E* (N/mm^2^)) was used as an index to express the hardness of the samples.

### 2.8. Color

The color of the white chocolate was measured using a MiniScan XE colorimeter (Hunter Associates Laboratory Inc., Reston, VA, USA) equipped with 4 mm measuring head. The colorimeter was calibrated with white and black reference standards. The CIE L* a* b* color scale was used, and the color parameters (L*, a* and b*) were measured at three different points on the chocolate surface. L* represents the lightness component, ranging from 100 (white) to zero (black), while a* (ranging from +redness to −greenness) and b* (ranging from +yellowness to −blueness) are the chromatic components. Color measurements were conducted in triplicate. Additionally, the Chroma (C*) values were calculated using the following equation:

(3) $$C^{*}=\sqrt{\left(a^{*2}+b^{*2}\right)},$$

### 2.9. β-Carotene Content in White Chocolate

The β-carotene content in white chocolate was determined using high performance liquid chromatography (HPLC). An HPLC Shimadzu HP 1100 Series (Columbia, MD, USA) equipped with a diode array detector and an automatic Agilent 1200 Series injector was used. The β-carotene was analyzed with a YMC C30 analytical column (5 µm, 250 × 4.6 mm I.D.) at ambient temperature, according to the experimental procedure described by Stramarkou et al. (2023) [26].

The isolation of β-carotene from white chocolate depended on its form, whether non-encapsulated or encapsulated. For WC-F, approximately 1.5–2 g of sample was ground and transferred into a test tube. Then, 3 mL of hexane was added, and the mixture was vortexed for 2 min. This was followed by centrifugation at 3000 rpm for 3 min, after which the supernatant enriched with β-carotene was removed. For white chocolate with incorporated encapsulated β-carotene, approximately 1.5–2 g of sample was ground and loaded into a test tube. First, 2 mL of water was added, and the mixture was vortexed for 2 min. Then, 2 mL of ethanol and 3 mL of hexane were added, and the mixture was vortexed again for 2 min. After centrifugation at 3000 rpm for 3 min, the supernatant enriched with β-carotene was collected. This process was repeated until the supernatant phase was completely decolorized. The β-carotene dissolved in the supernatant phase was then quantified using HPLC analysis.

### 2.10. Quality Characteristics of White Chocolates during Storage

The quality characteristics of white chocolates were evaluated during storage at 25 °C for 4 months. Thermal properties, rheological properties, hardness, color, and β-carotene content were determined at fixed time intervals (10, 20, 30, 45, 60, 75, 90, and 120 days) following the procedures described in Section 2.5, Section 2.6, Section 2.7, Section 2.8 and Section 2.9. The degradation data of β-carotene were fitted to a first-order reaction model as described in Equation (4):

(4) $$C=C_{o}\times e^{-kt},$$

where *C*_o_ is the initial concentration of β-carotene in white chocolate and *k* is the degradation constant rate.

### 2.11. Statistical Analysis

Statistical analyses were conducted using the STATISTICA^TM^ software package (version 12, StatSoft^®^ Inc., Hamburg, Germany). One-way analysis of variance (ANOVA) was applied to assess mean differences among groups. To further explore these differences, post hoc Tukey tests were performed, with a significance threshold set at *α* = 0.05.

## 3. Results and Discussion

### 3.1. Effect of Incorporated β-Carotene on the Quality Characteristics of White Chocolate

#### 3.1.1. Color

**Figure 1:**
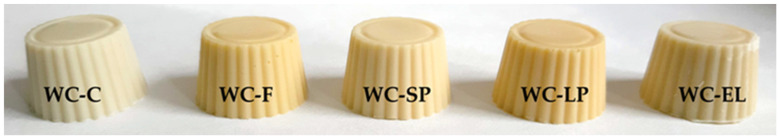
White chocolate samples.

The color is the most important characteristic of the appearance of foods and is a crucial factor for selecting and evaluating the quality of a food product. The impact of incorporating β-carotene, which is a lipophilic, red-orange pigment, on the color of white chocolate enriched with free and encapsulated β-carotene was studied. In Figure 1, the developed white chocolate samples are depicted.

**Figure 2:**
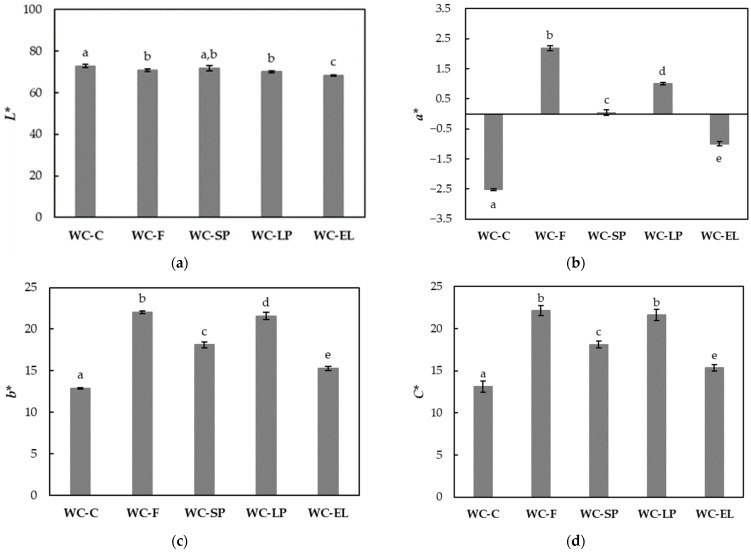
Color parameters (**a**) L*, (**b**) a*, (**c**) b*, (**d**) C* of the examined white chocolate samples. Bars with different letters indicate statistically significant differences (*p* < 0.05) between samples.

The color parameters L*, a*, and b* were determined for the white chocolate samples, and their values are presented in Figure 2. Based on the results, the lightness parameter (L*) of the white chocolate samples with free and encapsulated β-carotene showed small but significant differences compared to the control sample, except for the WC-SP. Additionally, the parameters a* and b* exhibited significant differences among the samples. The parameter a*, corresponding to red (+a*) and green (−a*) tones, increased from −2.52 (WC-C) to 2.18 (WC-F). Similarly, parameter b*, corresponding to yellow (+b*) and blue (−b*) tones, increased from 12.89 (WC-C) to 21.43 (WC-F). Therefore, the incorporation of free β-carotene imparts an intense red-orange color to white chocolate products, modifying their inherent characteristics. The encapsulation of β-carotene in polymeric structures emerges as an efficient strategy for mitigating the vibrant color associated with β-carotene. In direct comparison, the chromatic components (a* and b*) of WC-EL approached those of the control sample. WC-SP followed WC-EL, while WC-LP approached the values of WC-F, which included free β-carotene. This can be attributed to the porous structure of the lyophilized products, causing β-carotene migration into the chocolate mass, resulting in a color change. Mahfoudhi and Hamdi (2015) studied the color change in cake products after adding encapsulated and free β-carotene, reporting that the cake with free β-carotene was more intensely colored compared to the cake with the encapsulated ingredient [17]. Therefore, encapsulation is an effective method for masking the intense color of coloring agents.

The Chroma (C*) values for the different white chocolate samples, as depicted in Figure 2d, highlight significant variations among the formulations. The WC-C exhibited the lowest C* value, indicating the least color intensity. In contrast, the samples containing free β-carotene (WC-F) and lyophilized encapsulated β-carotene (WC-LP) demonstrated the highest C* values, reflecting more intense color characteristics. The WC-SP showed intermediate color intensity, while the WC-EL had the lowest color intensity among the encapsulated samples. These differences in C* values suggest that the encapsulation method significantly affects the color intensity of the white chocolate, with WC-LP and WC-F leading to the most pronounced color changes.

#### 3.1.2. Thermal Properties

**Figure 3:**
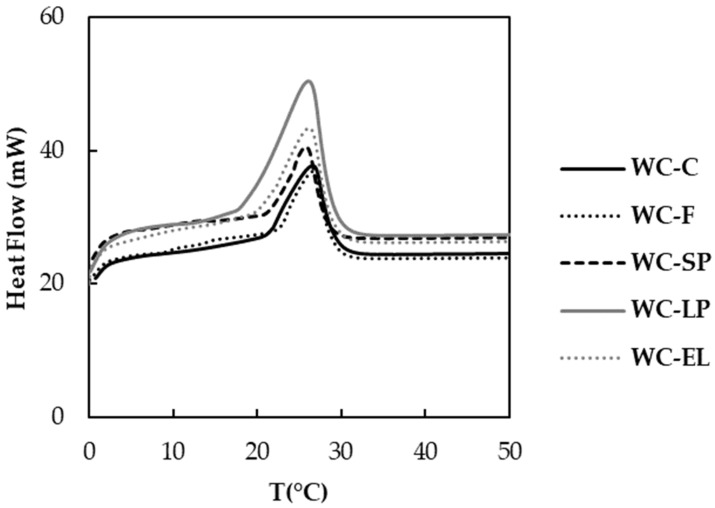
DSC plots for white chocolate samples.

The determination of chocolate’s thermal properties, especially its melting temperature, is crucial for evaluating its quality and is directly linked to its sensory attributes, reflecting how chocolate melts in the mouth. Figure 3 presents DSC plots for white chocolate samples. From the DSC scan, various parameters were identified, including the *T*_onset_ (marking the beginning of melting for a specific crystalline structure), *T*_peak_ (where the melting rate peaks), *T*_end_ (indicating complete melting), and the total energy required for melting (∆*H*_m_) [27,28]. These thermal properties are associated with the type of crystal into which cocoa butter crystallizes. Type V crystals are considered the most desirable as they melt close to body temperature (37 °C) [28].

The effect of incorporating free and encapsulated β-carotene on the thermal properties of white chocolate was evaluated. Table 1 presents the values of *T*_onset_, *T*_peak_, *T*_end_ and ∆*H*_m_ for the control sample and for the white chocolate enriched with the free and encapsulated β-carotene. According to the results, the onset, peak, and end temperatures ranged from 17.24 to 23.05 °C, from 25.79 to 26.60 °C, and from 30.25 to 31.31 °C, respectively, for the examined samples. The ∆*H*_m_ was determined to be within the range of 8.29 to 30.88 J/g. These values fall within the ranges reported in similar studies for white chocolate samples [29]. Based on statistical analysis, no significant differences were observed between the peak and end temperatures of the samples (*p* < 0.05). However, the onset temperature of samples with free and encapsulated β-carotene showed significant differences compared to the control sample (*p >* 0.05). The WC-LP sample exhibited a lower onset temperature compared to all samples, which can be attributed to the increased concentration of β-carotene lyophilized structures in the white chocolate mass. This is likely due to the thin layers of the fat phase covering a larger specific surface area of the particles in chocolate containing lyophilized structures. Similar observations have been reported by Didar (2020) and Lončarević et al. (2018), who noted a decrease in onset temperature in white chocolate samples containing encapsulated extracts of pomegranate and blackberry, respectively [10,30]. Finally, the WC-F and WC-SP samples presented lower melting energy compared to WC-C, while the WC-LP and WC-EL samples exhibited higher energy. Therefore, the WC-LP and WC-EL samples had a more compact structure compared to the other samples examined. This could be attributed to the increased concentration of lyophilized structures in WC-LP and PUL in WC-EL along with the crystallization of fats and the formation of a fat network in the chocolate [30].

#### 3.1.3. Rheological Properties

**Figure 4:**
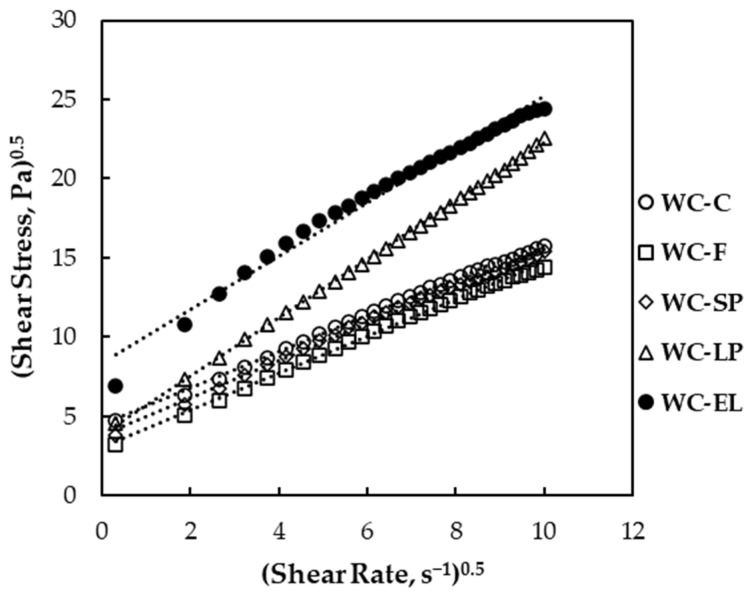
Plot of ($\dot{\gamma }$)^0.5^ versus (*σ*)^0.5^ for white chocolate samples that follow the Casson model.

A comprehensive understanding of rheological properties is essential for enhancing chocolate quality. The composition, fat content, emulsifiers, and solid particle distribution are key determinants that shape the rheology under distinct temperature and processing conditions [31,32]. The impact of incorporating free and encapsulated β-carotene on the rheological properties of white chocolate was evaluated in comparison to a control sample. Figure 4 illustrates shear stress as a function of shear rate for white chocolate samples. Rheological experimental data were fitted to the Casson model, showing a good fit for all samples (*R*^2^ > 0.98). Therefore, the incorporation of free and encapsulated β-carotene in white chocolate did not alter the non-Newtonian behavior of chocolate compared to the control sample.

**Figure 5:**
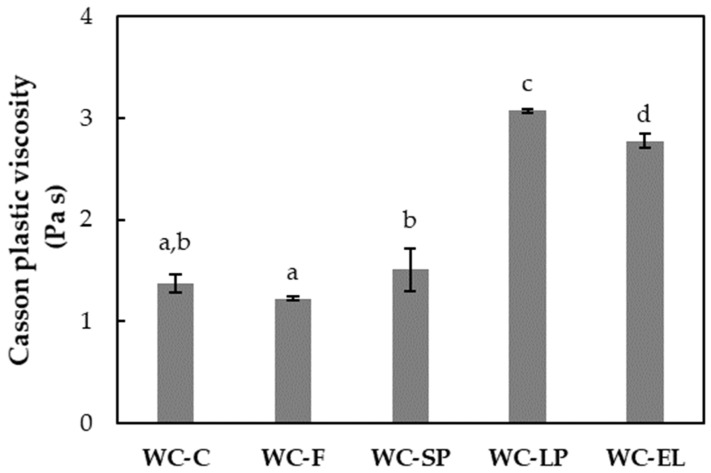
Plastic viscosity of the examined white chocolate samples. Bars with different letters indicate statistically significant differences (*p* < 0.05) between samples.

**Figure 6:**
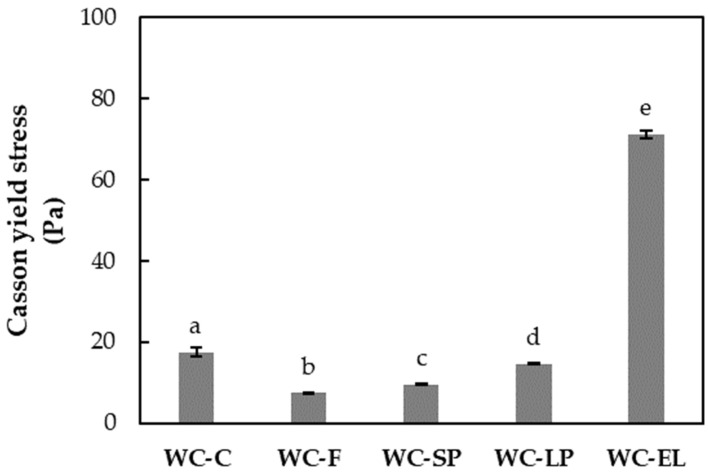
Yield stress of the examined white chocolate samples. Bars with different letters indicate statistically significant differences (*p* < 0.05) between samples.

In Figure 5 and Figure 6, the plastic viscosity and yield stress of the examined white chocolate samples are presented, respectively. Yield stress is related to the amount of energy required to initiate flow, while plastic viscosity expresses the amount of energy needed to maintain the flow [27]. The plastic viscosity of the samples ranged from 1.22 to 3.07 Pa s, and the yield stress varied from 7.37 to 71.15 Pa. Based on the results, the plastic viscosity of WC-F and WC-SP did not significantly differ from WC-C (*p* < 0.05). Conversely, WC-LP and WC-EL exhibited higher viscosity compared to WC-C, with WC-LP showing the highest viscosity among the samples. The increase in WC-EL viscosity can be attributed to the presence of PUL, which forms stable and viscous solutions. For WC-LP, the increase in viscosity is due to the augmented concentration of β-carotene lyophilized structures, which aim to achieve the desired β-carotene concentration, thereby reducing the free fat phase as the lyophilized structures overlap with cocoa butter and milk fats. Similar conclusions were reached by Lončarević et al. (2018) and Belščak-Cvitanović et al. (2012), who studied the rheological properties of chocolate enriched with encapsulated blackberry extract and plant polyphenols, respectively [10,11].

Examining the yield stress (Figure 6), notable distinctions were observed across all samples, with statistical significance compared to the control sample (*p* < 0.05). Particularly, WC-EL exhibited a remarkable elevation in yield stress, surpassing the other samples. In conclusion, the addition of encapsulated structures through the electrospinning process significantly influenced the rheological properties of white chocolate, which is mainly attributed to the composition of the structures. Notably, both plastic viscosity and yield stress increased for the WC-EL samples, which is not desirable during chocolate processing. Generally, the values of rheological parameters should be as low as possible to reduce the resistance of the chocolate mass during processing. Additionally, more fluid chocolate masses contribute to the development of improved organoleptic characteristics, such as increased perception of ‘smooth character’ during chewing [29].

#### 3.1.4. Hardness

Hardness is one of the most significant parameters frequently used to determine the texture of chocolate products. It is associated with the consistency and viscosity of molten chocolate, thereby influencing the organoleptic characteristics of chocolate [33,34]. Additionally, hardness serves as an indicator of tempering degree and the formation of the network of crystallized fats [35]. The hardness of chocolate products is mostly affected by their composition, particle size, tempering conditions, and temperature [36].

**Figure 7:**
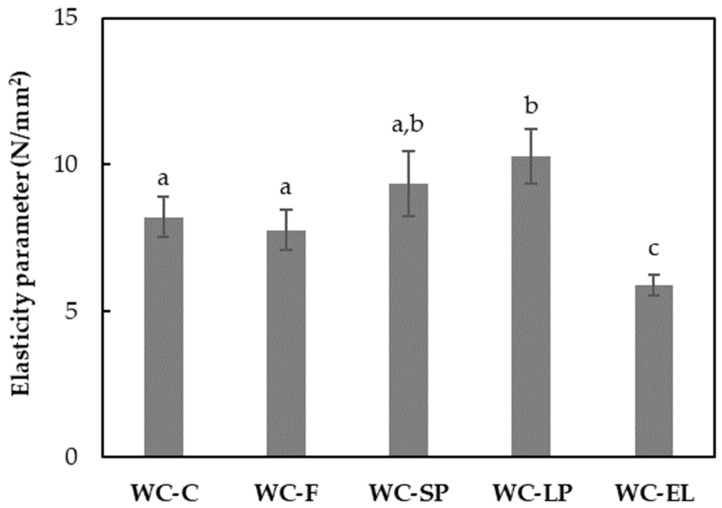
Elasticity parameter of the examined white chocolate samples. Bars with different letters indicate statistically significant differences (*p* < 0.05) between samples.

In Figure 7, the values of the elasticity parameter for white chocolate with encapsulated and free β-carotene are presented. The elasticity parameter values ranged from 5.89 to 10.28 N/mm^2^. Generally, a higher elasticity parameter value indicates greater hardness of the product. Based on the results, the hardness of WC-F and WC-SP did not differ significantly in comparison with the control sample (WC-C), in contrast to WC-LP and WC-EL. Notably, WC-LP showed slightly increased hardness compared to the control sample, while WC-EL exhibited significantly lower hardness. The increased concentration of β-carotene lyophilized structures in chocolate led to an increase in hardness, as protein tends to form bonds with other chocolate components, contributing to the formation of a network [35]. Conversely, the increased presence of PUL in white chocolate led to a reduction in hardness in the case of WC-EL. Similar observations were noticed by Genc Polat et al. (2020) and Lončarević et al. (2018), who reported an increase in hardness in white chocolate samples with the incorporation of encapsulated extracts from blackberry and pigment substances from the microalga *Nannochloropsis oculata*, respectively [10,13].

### 3.2. Quality Characteristics of White Chocolates during Storage

#### 3.2.1. Color

**Figure 8:**
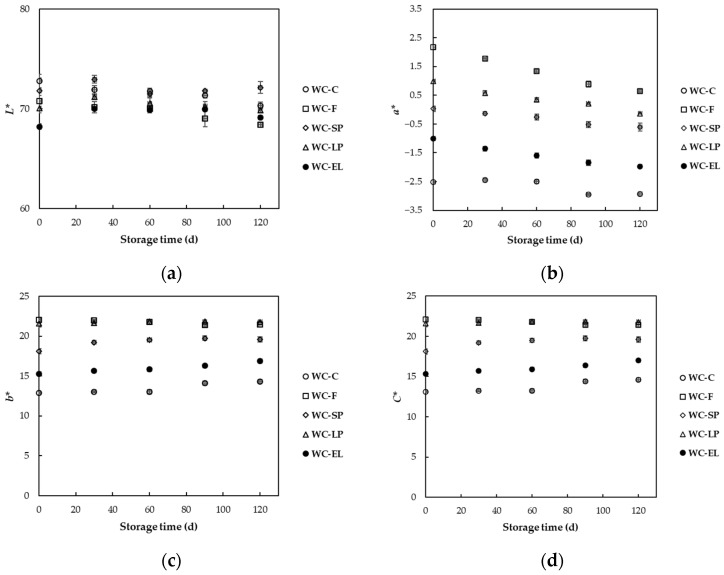
Color parameters (**a**) L*, (**b**) a*, (**c**) b*, (**d**) C* of white chocolate samples during storage at 25 °C.

In Figure 8, the variations in color parameters L*, a*, and b* during storage are presented. According to the results, the color parameters of the white chocolate samples changed significantly (*p* < 0.05) during storage. Specifically, the values of the color parameters a* and b* decreased with increasing storage time for all the examined samples. This phenomenon is attributed to the degradation of β-carotene through oxidation, resulting in the formation of colorless products [18]. More significant changes were observed in color parameter a*, with WC-SP, WC-EL, WC-LP, and WC-EL showing significantly lower values (−0.61, −1.97, −0.13, and 0.64, respectively) at the end of the storage period compared to their initial values (0.04, −1.00, 1.00, and 2.18, respectively). Similar findings were noted by Rocha et al. (2012), suggesting that, in some cases, evaluating just one of the chromatic components is adequate for assessing the color stability of a product during storage [37]. Notably, among the color parameters, the a* parameter exhibited variations in values 2–4 times greater than those observed for the L* and b* parameters during storage.

Moreover, the color parameters of white chocolate samples varied significantly (*p* < 0.05) depending on the method of incorporating β-carotene into the chocolate (encapsulated and non-encapsulated form). Among the studied encapsulated systems, the white chocolate enriched with β-carotene spray-dried structures (WC-SP) exhibited the best color stability, followed by WC-EL and WC-LP. Additionally, the incorporation of encapsulated β-carotene in white chocolate resulted in superior preservation of color stability compared to free β-carotene. Similar results were obtained by Coronel-Aguilera and San Martín-González (2015), who reported that encapsulated β-carotene structures resulted in increased color stability of yogurt during its storage for 4 weeks, showing minimal changes in the overall color of the sample [19]. In another study, Gomez et al. (2012) mentioned that the encapsulation of red pepper extract and its subsequent incorporation into a yogurt system led to a product with very stable color [38]. Furthermore, Jones et al. (2005) reported that lutein-enriched cheeses presented greater stability in color parameters compared to the control sample [39].

Figure 8d illustrates the changes in C* values of the white chocolate samples over a 120-day storage period. The C* values remained relatively stable throughout the storage period for all samples, with slight variations observed in some cases. The WC-C consistently showed the lowest C* values, indicating less intense color characteristics. In contrast, the samples with free β-carotene (WC-F) and lyophilized encapsulated β-carotene (WC-LP) exhibited similar, higher C* values, reflecting more intense color characteristics. The WC-SP displayed intermediate C* values, and the WC-EL consistently exhibited the lowest C* values among the encapsulated samples, indicating a lower color intensity. These results suggest that the choice of encapsulation method influences the color intensity of β-carotene in white chocolate during storage, with WC-F and WC-LP showing more intense color characteristics, while WC-EL provided a more stable and less intense color. The stability of the C* values over time also indicates that the color characteristics of the enriched white chocolate remain largely unaffected during storage, making these formulations viable for long-term use in functional foods.

#### 3.2.2. Thermal Properties

**Figure 9:**
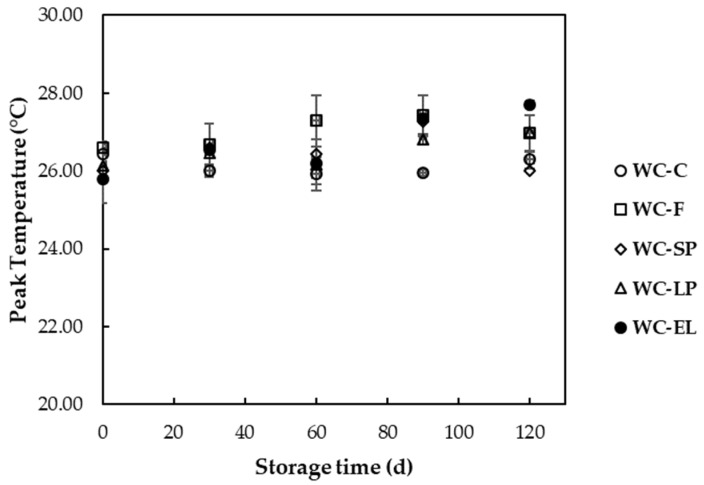
Peak (melting) temperatures of white chocolate samples during storage at 25 °C.

In Figure 9, the peak (melting) temperatures of white chocolate samples are presented during storage at 25 °C. According to the results, the melting point of the samples slightly changed, specifically increasing with storage. The slight increase in peak temperatures over storage time could be attributed to the gradual reorganization and stabilization of fat crystals within the chocolate matrix, enhancing the formation of more stable type V crystals. This process, also known as fat bloom, is commonly observed during the storage of chocolate products and can impact the overall quality and sensory attributes [40]. Similar studies have reported that the thermal properties of white chocolate are influenced by storage conditions and the presence of certain additives. For instance, a study by Rodriguez Furlán et al. (2017) demonstrated that storage temperature significantly affects the thermal properties and crystallization behavior of white chocolate. The study showed that different storage temperatures could lead to changes in the onset, peak, and end temperatures of melting, which are indicative of polymorphic transitions in cocoa butter [41]. Moreover, Ostrowska-Ligȩza et al. (2019) investigated the thermal and textural properties of milk, white, and dark chocolates, finding that the composition of the chocolate significantly affects its thermal behavior. They observed that the melting characteristics of chocolates depend on numerous factors, including the presence and quality of fat, sugar, and emulsifiers and particle size distribution [42].

#### 3.2.3. Rheological Properties

**Figure 10:**
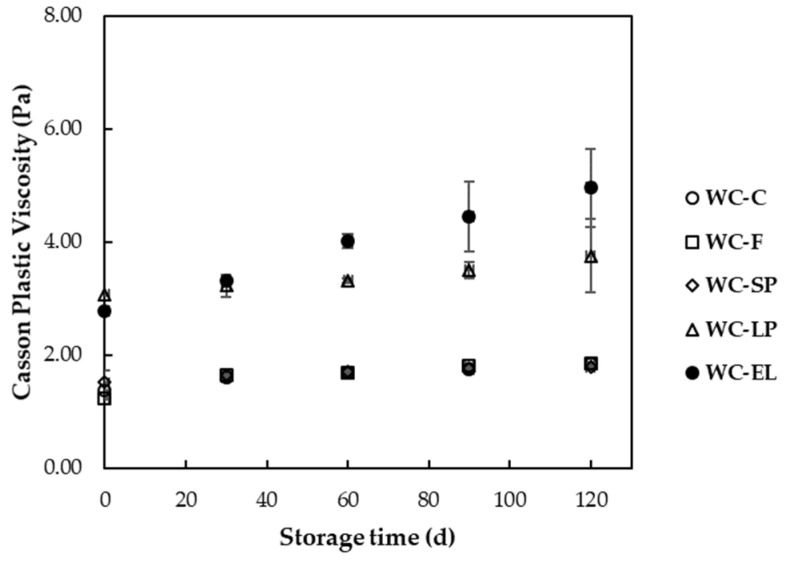
Plastic viscosity of white chocolate samples during storage at 25 °C.

**Figure 11:**
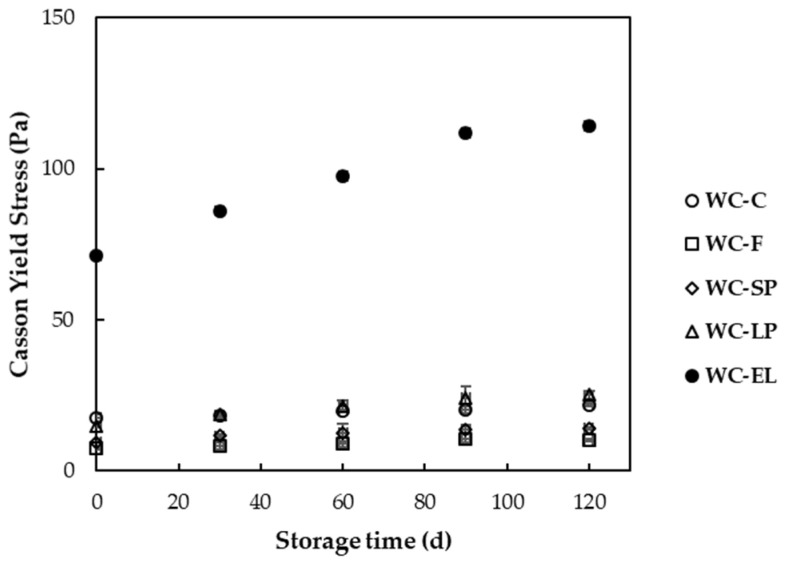
Yield stress of white chocolate samples during storage at 25 °C.

In Figure 10 and Figure 11, the changes in plastic viscosity and yield stress of white chocolate samples are depicted during storage at 25 °C. According to the results, the increase in storage time led to an increase in rheological parameter values for all the examined samples. These results can be correlated with the melting temperatures of the samples, which exhibited an increasing trend with storage, leading to difficulties in the flow of chocolate during melting. The observed increase in plastic viscosity and yield stress over time can be attributed to the stabilization of more stable fat crystal forms, such as type V and type VI crystals. These crystals are known to be more resistant to deformation, thereby increasing the viscosity and yield stress of the chocolate. Furthermore, the phenomenon of fat bloom, which involves the migration and recrystallization of fat on the surface of the chocolate, can also contribute to changes in rheological properties during storage [43]. Studies have shown that the rheological properties of chocolate are significantly affected by storage conditions. Bhattacharyya and Joshi (2022) observed that chocolate undergoes changes in rheological behavior due to solid–liquid transitions influenced by temperature and mechanical stresses. Their study highlighted that these transitions can affect the chocolate’s consistency and flow properties [44]. Additionally, the study by Vásquez et al. (2019) indicated that the composition of chocolate, including fat content and particle size distribution, plays a crucial role in its rheological properties. They found that the degree of chocolate structuring is a function of the solids content, and most rheological properties significantly depend on them [45].

#### 3.2.4. Hardness

**Figure 12:**
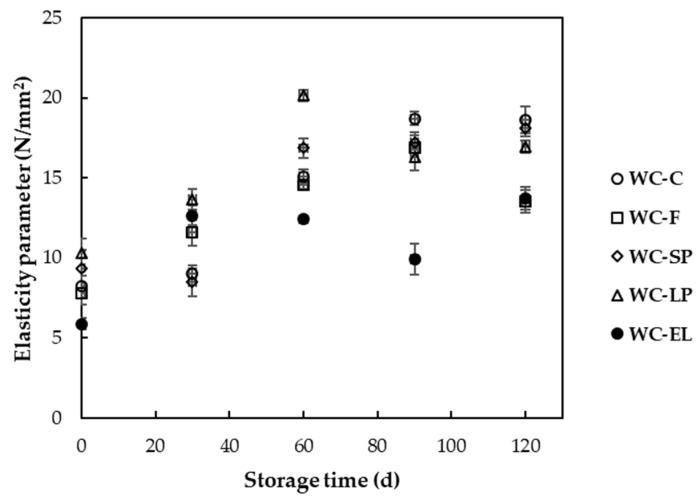
Elasticity parameter of white chocolate samples during storage at 25 °C.

In Figure 12, the changes in hardness of white chocolate samples are illustrated during storage at 25 °C. Based on the results, it was observed that the hardness of the samples increased with increasing storage time. This trend has been observed in other studies of chocolate as well [46]. The increase in hardness during storage can be attributed to the transition of the crystal type from IV and V (according to the melting temperature) to more stable structures. Studies have shown that storage conditions significantly affect the textural properties of chocolate. For example, Bhattacharyya and Joshi (2022) found that changes in storage temperature and duration can lead to alterations in the hardness and other textural properties of chocolate. Their study highlighted the importance of controlling storage conditions to maintain the desired texture of chocolate products [44]. Similarly, Nafingah et al. (2019) investigated the impact of fat content and palm sap sugar proportion on the heat stability and hardness of milk chocolate. They found that increased fat content generally decreased the hardness of chocolate, whereas higher proportions of palm sap sugar, due to its moisture content, increased hardness. These findings suggest that compositional changes during storage can influence the hardness of chocolate [47].

#### 3.2.5. Degradation Kinetics of β-Carotene during Storage

**Figure 13:**
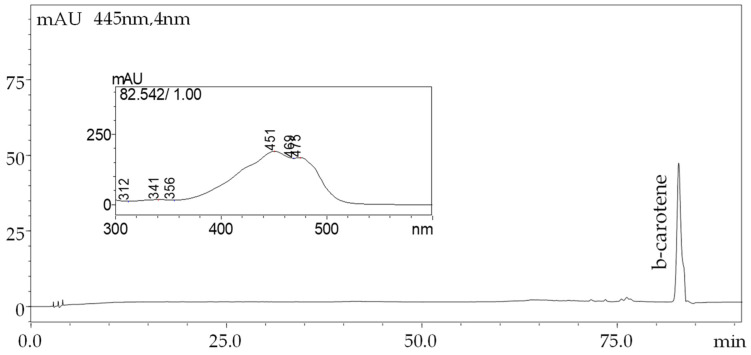
HPLC chromatogram of β-carotene in white chocolate samples. The UV-visible absorption spectrum of β-carotene peak from HPLC analysis is given in the inset.

Figure 13 shows the HPLC chromatogram for the analysis of β-carotene in the white chocolate samples. The chromatogram displays a clear peak corresponding to β-carotene, confirming its presence and successful encapsulation within the chocolate matrix. The retention time observed for β-carotene was consistent with standard references, ensuring accurate identification. This chromatographic analysis was crucial for quantifying the β-carotene content and assessing its stability across different encapsulation methods used in this study. The HPLC method provided precise separation and quantification, reinforcing the reliability of the data. From Figure 13-inset, the UV-visible absorption spectrum corresponding to the β-carotene peak identified in the HPLC analysis is displayed, ensuring that the peak identified corresponded specifically to β-carotene. The three absorption maxima observed at 451 nm, 469 nm, and 475 nm align precisely with the known spectral characteristics of β-carotene, which typically exhibits these peaks due to its extended system of conjugated double bonds [14]. The presence of all three characteristic peaks in the spectrum provides robust evidence that the compound isolated and quantified during HPLC is indeed β-carotene, thereby supporting the reliability of the chromatographic data presented in this study. Such spectral confirmation is essential when analyzing complex food matrices, as it rules out the possibility of co-eluting compounds interfering with the β-carotene peak [15].

**Figure 14:**
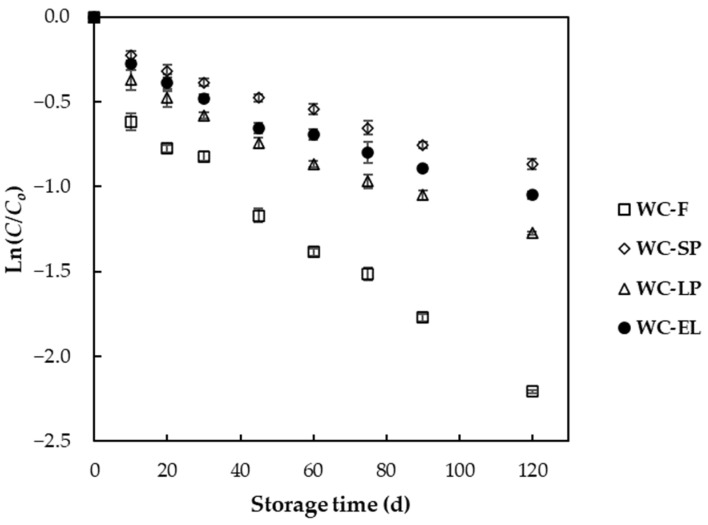
First-order degradation kinetic plots for white chocolate samples during storage at 25 °C.

The degradation data kinetic plots of free and encapsulated β-carotene through the electrospinning process, spray drying, and freeze drying in white chocolate are presented in Figure 14 during storage at 25 °C.

The experimental β-carotene degradation data were fitted to a first-order reaction model, and the values for the degradation rate constant (*k*), half-life periods (*t*_1/2_) and regression coefficients were obtained through nonlinear regression (Table 2). According to the results, the first-order reaction model described the experimental data for all the examined samples quite well, yielding good fits with *R*^2^ values exceeding 0.93, as reported in other studies related to the oxidative degradation of β-carotene [48]. Additionally, a study by Demiray and Tulek (2017) found that β-carotene degradation in carrot slices during convective drying followed a first-order reaction model, with temperature significantly affecting the degradation rate. This supports the finding that the first-order reaction model can effectively describe the degradation kinetics of β-carotene in various food systems [49]. Among the examined samples, WC-SP had the longest half-life period, followed by WC-EL and WC-LP. Comparatively, the incorporation of β-carotene into carriers increased the half-life period by 2, 1.6, and 1.8 times for WC-SP, WC-LP, and WC-EL, respectively, compared to WC-F, signifying encapsulation as an effective preservation method during storage [50].

When comparing the efficiency among the developed encapsulated structures, it was observed that the incorporation of lyophilized and electrospun structures into white chocolate led to lower β-carotene stability during storage. This outcome could be attributed to the porous structure of lyophilized products and possibly the polymeric composition of structures developed through electrospinning. Notably, structures with increased PUL concentration did not effectively protect β-carotene compared to SP structures (80% wt. WPI), suggesting that the composition of the encapsulating material plays a crucial role in preserving the stability of the encapsulated compound. These results align with previous findings by Ezhilarasi et al. (2013), who reported similar trends in the stability of citric acid during baking, where microcapsules with whey protein provided superior protection compared to maltodextrin or a mixture of both [51].

In conclusion, encapsulation significantly enhances the stability of β-carotene in white chocolate, with spray-dried structures showing the highest preservation efficiency. The composition and structure of the encapsulating material play a critical role in determining the effectiveness of β-carotene protection during storage.

## 4. Conclusions

This study successfully developed functional white chocolate enriched with β-carotene using three different encapsulation techniques: spray drying, freeze drying, and coaxial electrospinning. These techniques were evaluated for their impact on the quality characteristics of white chocolate and the stability of β-carotene during a 4-month storage period at 25 °C. The results demonstrated that the spray drying (WC-SP) method provided superior β-carotene retention, with a degradation rate constant (k) of 0.0066 day^−1^ and a half-life of 126.04 days, which is twice the half-life of free β-carotene (WC-F). The coaxial electrospinning (WC-EL) and freeze drying (WC-LP) methods also showed significant benefits, with degradation rate constants of 0.0080 day^−1^ and 0.0094 day^−1^, respectively, indicating better stability compared to the free β-carotene. In terms of thermal properties, no significant differences were found in the melting profile temperatures among the samples, but WC-LP and WC-EL exhibited higher melting energies (30.88 J/g and 16.00 J/g, respectively) compared to the control (12.42 J/g), suggesting a more organized structure. Rheological and hardness properties varied, with WC-LP showing slightly increased hardness (10.28 N/mm^2^) and WC-EL showing significantly decreased hardness (5.89 N/mm^2^), highlighting the influence of encapsulation on textural characteristics. Furthermore, color stability was best preserved in WC-SP, followed by WC-EL, indicating the effectiveness of these methods in maintaining the appearance of the chocolate. The findings of this study highlight the potential of spray drying and coaxial electrospinning as promising techniques for enhancing the stability and quality of bioactive-enriched confectionery products. The novelty of using coaxial electrospinning, in particular, offers a novel approach to improving the stability of sensitive bioactives in food matrices. While the study provides valuable insights into the stability and quality enhancement of β-carotene-enriched white chocolate, a limitation is the lack of investigation into the bioavailability of the encapsulated β-carotene. Future research should address this gap by exploring the bioavailability of encapsulated bioactives in such food matrices. Additionally, further studies should explore the scalability of these methods for industrial applications and investigate the sensory characteristics and consumer acceptance of these functional products.

## Figures and Tables

**Table 1:** Effect of incorporated free and encapsulated β-carotene on the thermal properties of white chocolate.

	WC-C	WC-F	WC-SP	WC-LP	WC-EL
β-carotene concentration in white chocolate	1.0 mg/100 g white chocolate				
Mass of encapsulated β-carotene structures /total mass of white chocolate fortified with encapsulated structures (% (*w*/*w*))	0	0	0.67	7.50	2.67
Onset temperature (°C)	21.79 ± 0.77 ^a^	23.05 ± 0.32 ^b^	23.58 ± 0.77 ^b^	17.24 ± 0.15 ^c^	20.77 ± 0.24 ^d^
End temperature (°C)	31.08 ± 0.05 ^a,c^	31.31 ± 0.10 ^a^	30.25 ± 0.14 ^b^	31.32 ± 0.05 ^a^	30.63 ± 0.72 ^b,c^
Peak temperature (°C)	26.43 ± 0.32 ^a^	26.60 ± 0.08 ^a^	26.02 ± 0.04 ^a,b^	26.15 ± 0.03 ^a,b^	25.79 ± 0.61 ^b^
Enthalpy required for melting of chocolate (Δ*H*, J/g)	12.42 ± 1.18 ^a^	9.68 ± 1.55 ^b^	8.29 ± 0.58 ^b^	30.88 ± 1.94 ^c^	16.00 ± 0.53 ^d^

On each row, columns with different letters significantly differ (*p* < 0.05).

**Table 2:** Degradation constant rates (*k*), half-life periods (*t*_1/2_) and correlation coefficients for the examined white chocolate samples.

Sample	*k*·10^−3^ ± *s*k·10^−3^ (d^−1^)	*t*1/2 (d)	*R* ^2^
WC-F	16.4 ± 1.6 ^a^	61.95	0.95
WC-SP	6.6 ± 0.6 ^d^	126.04	0.95
WC-LP	9.4 ± 0.2 ^b^	98.33	0.93
WC-EL	8.0 ± 0.4 ^c^	109.51	0.93

Values labeled with different letters indicate significant differences (*p* < 0.05).

## Data Availability

The original contributions presented in the study are included in the article, further inquiries can be directed to the corresponding author.
